# Cross-Species Gene Expression Analysis of Species Specific Differences in the Preclinical Assessment of Pharmaceutical Compounds

**DOI:** 10.1371/journal.pone.0096853

**Published:** 2014-05-13

**Authors:** John Okyere, Ekow Oppon, Daniel Dzidzienyo, Lav Sharma, Graham Ball

**Affiliations:** 1 CrossGen Limited, BioCity Nottingham, Pennyfoot Street, Nottingham, United Kingdom; 2 John Van Geest Cancer Research Centre, Nottingham Trent University, Clifton Campus, Clifton Lane, Nottingham, United Kingdom; Taipei Medical University, Taiwan

## Abstract

Animals are frequently used as model systems for determination of safety and efficacy in pharmaceutical research and development. However, significant quantitative and qualitative differences exist between humans and the animal models used in research. This is as a result of genetic variation between human and the laboratory animal. Therefore the development of a system that would allow the assessment of all molecular differences between species after drug exposure would have a significant impact on drug evaluation for toxicity and efficacy. Here we describe a cross-species microarray methodology that identifies and selects orthologous probes after cross-species sequence comparison to develop an orthologous cross-species gene expression analysis tool. The assumptions made by the use of this orthologous gene expression strategy for cross-species extrapolation is that; conserved changes in gene expression equate to conserved pharmacodynamic endpoints. This assumption is supported by the fact that evolution and selection have maintained the structure and function of many biochemical pathways over time, resulting in the conservation of many important processes. We demonstrate this cross-species methodology by investigating species specific differences of the peroxisome proliferator-activator receptor (PPAR) α response in rat and human.

## Introduction

Comparative transcriptomics aims to understand organism diversity and the conservation of phenotypic responses across species. Conserved sequences between species are referred to as orthologs. These are sequences that evolved from a common ancestral gene and have retained the same function during the course of evolution. As a consequence animal models are frequently used in toxicology to assess the potential effects of a chemical on humans. This necessitates the use of comparative transcriptomics tools [1]–[4] (to determine whether an adverse response observed in a model species is conserved in humans. Although the magnitude of responses may differ between model species and human, if these differences are consistent, extrapolation of data to human is valid [5].

One major drawback to the concept of cross-species extrapolation is species specific response to chemicals. This is when the adverse response to chemical exposure is not conserved between model species and humans. Comparative transcriptomic approaches of gene expression changes for human and model species can identify divergent sets of genes specific to human and the model species while pathway enrichment analysis of divergent cross-species gene expression changes can confirm sensitivity of organ toxicity to humans [6], [7].

A number of drugs, pesticides, plasticizers, industrial chemicals and specific diets cause pleitropic effects including proliferations of peroxisomes. These pleitropic effects are mediated by activation of the peroxisome proliferator-activator receptor (PPAR) α. Short term studies of the effects of rodent peroxisome proliferators have been conducted in many laboratories resulting in the identification of marked species differences in response [8]–[10].

Rats and mice are clearly responsive to peroxisome proliferators whereas the guinea-pig and dog are either unresponsive or refractory. Peroxisome proliferation in primates is greatly reduced compared to rodents [8]. The ability of PPAR agonists to cause human liver peroxisome proliferation in vivo is mixed. Human and rat PPARα have similar functions. There is also a high homology of their DNA and ligand binding domains [11]. However, there is a marked decrease in PPARα expression of human hepatocytes in the presence of an agonist. Ammerschlaeger et al [11] showed that these species differences are due to differences in the promoter response elements of target genes. They also observed that human hepatocytes limit the activity of PPARα.

PPARα functions as a ligand-inducible transcription factor for genes involved in mitochondrial and peroxisomal metabolism. Marked species differences in response to peroxisome proliferators exist where rodents show high peroxisomal enzyme induction while humans do not [12]. One factor accounting for this species specificity is the responsiveness of PPARα regulated genes that is defined by PPAR response elements (PPRE) located within the promoter region of target genes. PPRE has recently been found in the human C3 promoter of the complement system in the liver [13] and has been shown to be conserved between mouse and human, suggesting a regulatory mechanism possibly common with PPAR alpha targets across species.

Over expression of PPARα in human hepatocytes to the levels comparable to those observed in rat primary hepatocytes does not increase the induction of peroxisomal activity, suggesting that differences in receptor levels alone cannot account for a lack of peroxisomal activity [11]. Ammerschlaeger et al [11] also demonstrated that transient transfection assays with the PPARα agonists, “ciprofibrate and Wy 14,643 induced rat but not human PPARα-mediated reporter gene activity”. Their results further showed that “human hepatocytes limit the extent of peroxisome proliferation regardless of PPARα expression”.

Preclinical studies of PPARα agonists usually do not predict hepatoxicity to humans. This is perhaps PPARα agonist may regulate different sets of genes in rodents and humans. As a consequence, in the past, human liver cells have been used rather than rodent liver cells to investigate hepatoxicity of PPARα agonists to humans. However, primary human hepatocyte cultures are limited by inter-individual variability and short term in vitro life span which does not allow long term study of PPAR agonists in primary human hepatocyte cultures [9].

The cell culture medium of human hepatocytes used for investigating the PPARα response may have a limiting effect on hepatocyte gene expression. Reports [14] show that in contrast to the in vivo situation where the liver maintains its differentiated functions while hepatocytes are undergoing cellular proliferation following toxic damage, proliferation and differentiation in hepatocytes are inversely related in vitro [15] where proliferation leads to dedifferentiation of hepatocytes. Although it is clear that varying culture conditions can have profound effects on the transcription of liver-specific genes, many differentiated functions are lost regardless of the culture conditions [16]–[18].

Therefore an ideal or close to ideal system characterised by the long term expression of all liver specific functions at the in vivo level, which could be used for long term in vitro studies such as chronic hepatoxicity, could advance the comparative transcriptomic analysis of chemicals intended for human use.

Studies of cross-species interactions have attempted to identify orthologous sets of genes as differentially expressed genes common to human and model species after species specific microarray analysis of human and model species samples [6], [7]. This approach does not allow for the investigation of specific mechanism of activity of the putative sets of orthologous genes since they cannot be separated from the other species specific sets of genes. This could be problematic in the identification of gene expression profiles that could be diagnostic of a toxicity endpoint.

The multiple probes per transcript feature of the Affymetrix GeneChip microarray platform enables the selection of a subset of probes for the analysis of transcripts. It is therefore possible to re-map GeneChip probes by sequences comparison to generate new probe set definitions to interrogate the transcriptome [19]. For cross-species analysis this approach is powerful in that it allows for the computation of new expression estimates for the orthologous transcripts.

This study was aimed at investigating the utility of a cross-species gene expression strategy in an attempt to characterise the species specificity of PPARα activity and to demonstrate that the weak PPARα response of human hepatocytes is partly due to the primary hepatocyte culture environment. We used data generated previously [18] and employed a cross-species gene expression analysis strategy to identify human orthologs of rat Affymetrix GeneChip probes. The human/rat cross-species orthologous probes were used to compute new expression estimates for human, in an attempt to mimick the wild type in vivo phenotype of the human hepatocyte environment.

## Materials and Methods

### Total RNA Preparation and Analysis

All animal experiments were performed by Merck KGaA (Darmstadt, Germany). Details of the animal and human samples used to generate the microarray data for the work was published by Merck Serono [18].

The animal experiments were approved by the local animal welfare committee, Hessian Regierungspraesidium, and were conducted with the principles of Good Laboratory Practice of the Organization for Economic Cooperation and Development, the European Union, and the Food and Drug Administration Good Laboratory Practice regulation 21, Code of Federal Regulations Part 58, as well as the local animal welfare regulations. All experimental procedures regarding human samples were performed in compliance with French law and regulations after the approval of the National Ethics Committee (Paris, France). Informed written consent was obtained from all patients for use of samples for research purposes.

Total RNAs for this study were extracted from rat and human hepatocytes isolated from liver samples by Richert et al (2008). Rat hepatocytes from each animal were treated with 30 µM EMD, 100 µM or 0.1% DMSO as vehicle control at 24 hrs and 72 hrs. Three biological replicates were prepared for each time point at each concentration of EMD 392949 and DMSO giving a total of 18 rat samples. The same treatment regime was applied to the human hepatocytes.

Total RNA was isolated with TRI reagent following manufacturer’s protocol. Quality and concentration of RNA were determined using the NanoDrop spectrophotometer and the Agilent Bioanalyzer 2100 applying the total RNA Nano Assay following the manufacturer’s protocols.

### Microarray Hybridization

10 mg of total RNA, obtained from the work of Richert et al [18], was converted to double-stranded cDNA, followed by cRNA synthesis, hybridization, washing, and scanning using standard microarray processing protocols; GeneChip analysis was performed according to the Affymetrix manufacturer’s protocol (Affymetrix Genechip Expression Analysis, Technical Manual). Rat hepatocyte samples were hybridized to Rat Genome 230 2.0 GeneChips and Human Genome U133 Plus 2.0 GeneChips were utilized to analyze RNA obtained from human hepatocytes. All microarray data were submitted to the NCBI Gene expression Omnibus as a series with the accession number GSE47972.

Downstream analyses was performed with the RMA [20] condensed expression estimates using GeneGo MetaCore and supervised learning strategy based on an artificial neural networks (ANNs) modelling approach. ANNs are a form of artificial intelligence that can model complex systems and have shown good predictive performance on blind data [21], [22].

### GeneChip Probes

Affymetrix uses mRNA sequences obtained from public databases and clusters them to 90% sequences identity. The longest sequence in each cluster is used as the representative for that cluster, with preference given to RefSeq sequences. Pairwise alignment of probe sequences against the non-redundant mRNA set is used to assign probe sets to transcripts. There are several probes per probe set [23]. A probe set usually represents a gene. Each probe is 25 bases long grouped in pairs. Each probe pair consists a perfect match (pm) probe, designed to match perfect a target gene sequence, and a mismatch (mm) probe, designed to measure non-specific hybridization. The mismatch probe differs from its associated perfect match probe only in the 13^th^ base.

### Orthologous Probe Selection

GeneChip Rat Genome 230 2.0 probe sequences were obtained from Affymetrix (www.affymetrix.com) to perform sequence comparisons with human sequences using BLAST [24]. The following steps were carried out computationally;

- A local human cDNA database was generated with sequences sourced from the National Center for Biotechnology Information (NCBI). Each perfect match 25 mer Rat GeneChip probe queried the human cDNA database using a word size of 25.
- The returned results were collected and screened for probe sequences with 100% matches. In some cases not all selected rat probes had 100% matches with a human cDNA. These were defined as probes that had at least 20 bases matching human cDNA bases in tandem.
- Probes were also selected that had one or two mismatch base at the end of the 25 mer probe.
- To ensure the selection of gene specific probe sets, probes with multiple matching cDNA sequences were eliminated. However, as explained above, not all rat probes were 100% matches to their corresponding human cDNA sequences. It is noteworthy that sometimes cross hybridizing probes can be useful since the cross hybridizing transcript is usually not expressed at a level that leads to signal interference in a given tissue or sample. Furthermore, probe level analysis algorithms such dChip [25], RMA [20] and PLIER [26] are capable of identifying cross-hybridizing probes and eliminating these probes from the computation of the expression estimates.
- In some cases where probes detected several reference sequences, most of the results were manually evaluated to select suitable probes outside the stated criteria. For example, where all the probes in a probe set matches 100% to multiple reference sequences, probes can be manually identified where fewer than 10 mismatch bases occur at the ends of the 25 mer probe.
- All other probes intermingling with probes mapping to a different cDNA reference sequence are eliminated. This results in fewer probes for most of the selected probe sets but with greater specificity.
- Probe sets containing at least three probe pairs were used to construct a new library file to analyse rat RNA samples as human. This new cross-species rat/human orthologous library file represents a ‘virtual’ human ‘microarray’ capable of analysing rat transcriptome as human.
- A minimum of three probe pairs per probe set should satisfy [19] the minimum requirement for most probe level analysis algorithms. The reduced number of probes leads to a slight drop in statistical power for each probe set. However, this is compensated by the high specificity of the orthologous probe sets.
- A new library file of 31004 orthologous rat/human cross-species probe sets, 96 probe sets less than the Affymetrix rat GeneChip array, was constructed following the probe selection criteria described above. Construction of the new library file with the selected orthologous probes, computationally, takes almost a fortnight of program run time.
- The multiple probes per probe set feature of the Affymetrix GeneChip microarray platform allows for robust selection of orthologous sets of probe sets for cross-species comparison. To this end, Affymetrix have designed spreadsheet datasets containing cross-species information on orthologous probes between human, rat, mouse, (www.affymetrix.com/support/technical/comparison_spreadsheets.affx).
- The methodology described here builds on the Affymetrix cross-species comparison approach described by the spreadsheets and develops new library files containing cross-species orthologous probes. The advantage of this approach is that, the probe selection methodology allows for the computation of new gene expression estimates, unlike the Affymetrix spreadsheets.

There is a significant number of probe set redundancy with GeneChips in a one-to-many relationship with the target sequence [27]. This can be very problematic for downstream analysis, thus requiring re-mapping of GeneChip probes to aide better interpretation of GeneChip data. In this cross-species study we have mapped probes on a one-to-one relationship between rat and human. To select orthologous rat probes using human as the reference genome, a subset of the 11 perfect match GeneChip Rat Genome 230 2.0 probes, were identified for each orthologous transcript. Probe sets with less than three orthologous probes were excluded from library file construction.

### Analysis with Cross-species Library File

The rat CEL file data, generated by hybridising RNA from cultured rat hepatocytes challenged with EMD 392949 (EMD), were analysed with the rat/human cross-species library file (virtual human). Using the RMA algorithm, new expression estimates were generated with the cross-species library file. The following steps were carried out computationally;

- Using a text editor the header information of each Affymetrix rat 230.2 array CEL file, hybridised with rat hepatocyte challenged with EMD, was amended to include the file name of the cross-species library file. Thus, the Affymetrix rat 230.2 array CEL files were made compatible with the cross-species library file.
- The cross-species library file was placed in a folder accessible by Affymetrix Expression Console application.
- The library path of the Affymetrix Expression Console application was redirected to access the folder containing the cross-species library file.
- The Affymetrix rat 230.2 CEL files, amended with the cross-species library file header information, was uploaded into Expression Console application.
- Expression analysis was performed with Affymetrix Expression Console application using the RMA algorithm workflow.
- The new expression estimates (virtual human), generated with the cross-species library file, for the Affymetrix rat 230.2 CEL files, were exported into Excel files for downstream analysis.
- Real rat and real human expression data for comparison with virtual human were generated following standard Affymetrix Expression Console application protocol.

The new cross-species data generated with the rat samples (converted Affymetrix rat 230.2 CEL files) represents a virtual human hepatocyte transcriptome.

### Pathway Enrichment Analysis

The enrichment analysis was conducted using GeneGo pathway maps in the Metacore database (Version 6.4 build 26113; GeneGo, St. Joseph, MI). A hypergeometric distribution strategy was used to calculate the enrichment p-values using the GeneGo database as background. Gene expression estimates were computed using the Robust Multi-array Average (RMA) algorithm with a log base 2 transformation [20]. Fold change values for differentially expressed genes were computed and used for GeneGo pathway mapping and pathway enrichment analysis. A false discovery correct p-value<0.05 was defined as significant for the enrichment analysis.

## Results and Discussion

### Increased Activity of PPARα Markers by Orthologous Probe Selection

**Figure 1:**
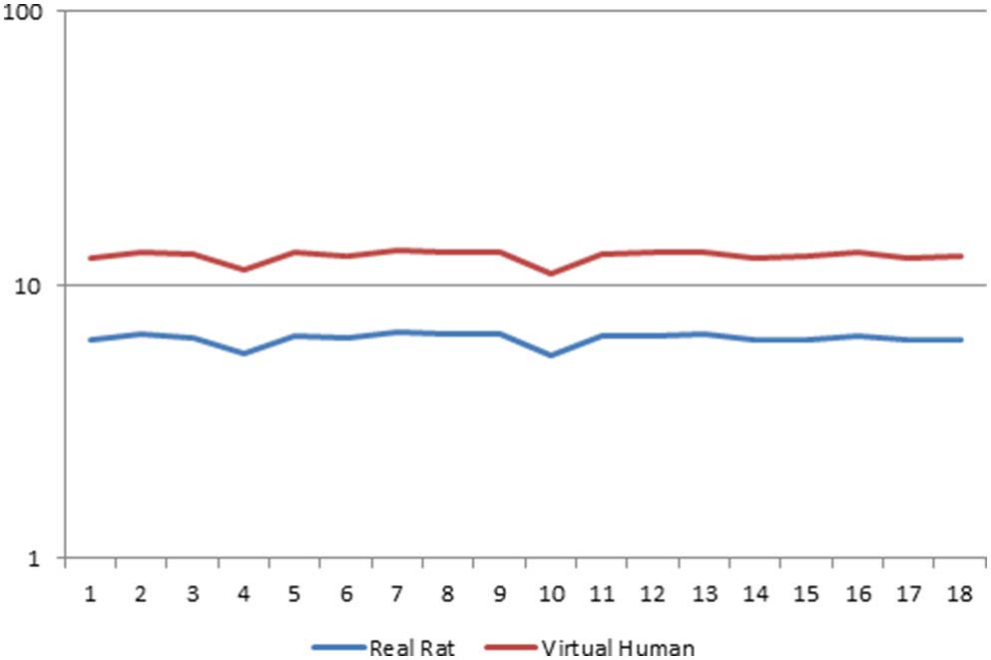
Comparison of PPARα gene expression profile between real rat and virtual human. Gene expression profile of PPARα gene (1387278_at) activity on y-axis for ‘virtual’ human and real rat in 18 samples (x-axis). The RMA condensed expression estimates were computed with ‘virtual’ human (Rat/Human cross-species library file) and real rat (Affymetrix Rat 230.2 array) library files.

PPARα mediated responses involve regulation of lipid metabolism, peroxisomal proliferation and the induction of growth regulatory genes. Figure 1 depicts the effects of EMD, a PPARα/PPARg dual agonist [18], on the gene expression profile of the 18 samples. PPARα marker enzyme activity like acyl CoA oxidase (ACOX) was highly expressed in both real rat and ‘virtual’ human. However, human hepatocytes display only weak induction of ACOX [11].

**Figure 2:**
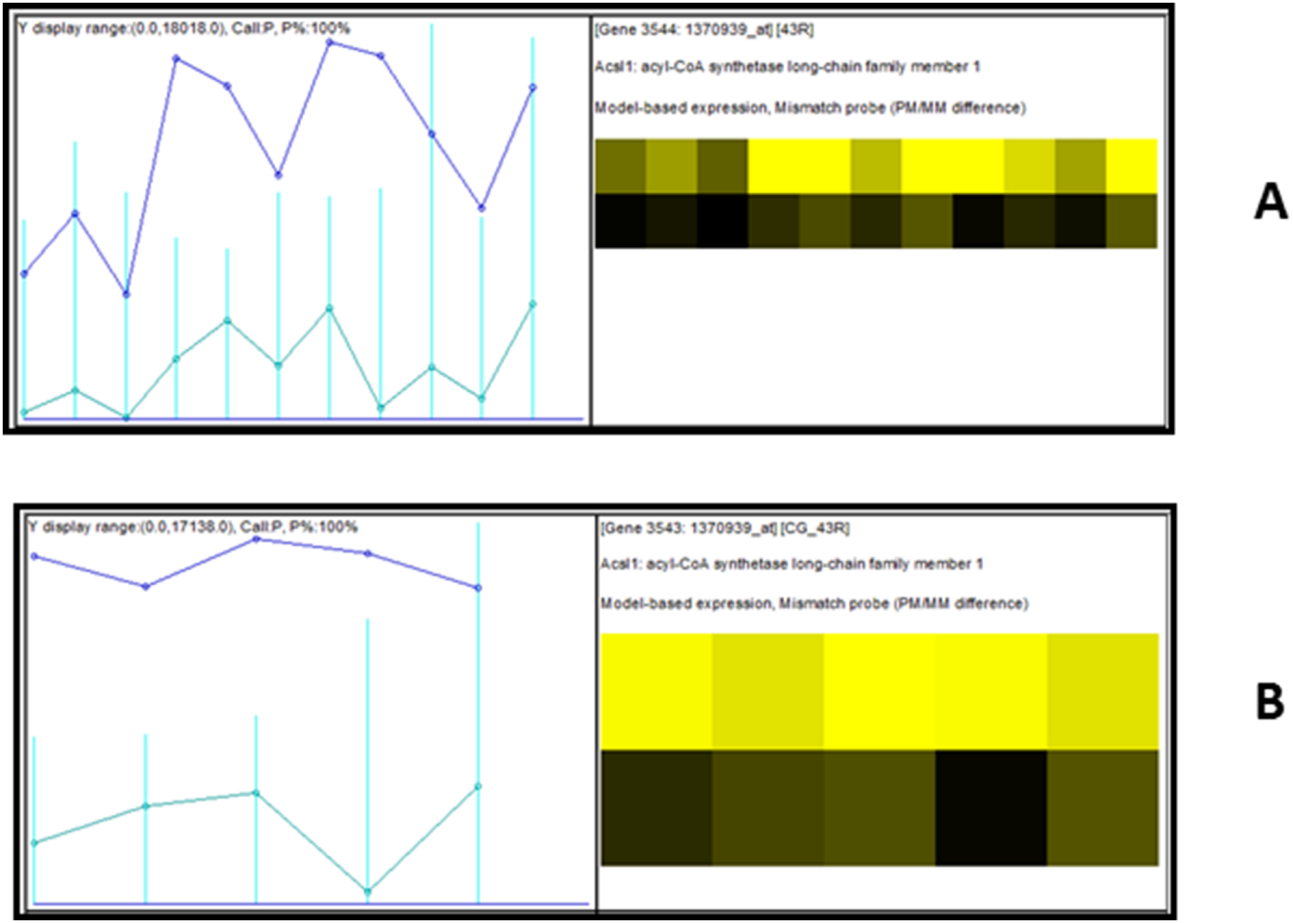
Graphical representation of PPARα probe intensity response. Probe intensity profiles for real rat (**A**) and virtual human (**B**) arrays. Informative probes from Affymetrix Rat230.2 array were identified and selected to construct the ‘virtual’ human cross-species library file after comparative genome sequence analysis. The probe intensity profiles demonstrate that sensitivity of the ‘virtual’ array was significantly improved over the sensitivity of the reference Affymetrix rat230.2 array. The ‘virtual’ human cross-species array consists of human ortholougs of Affymetrix rat230.2 probes.

PPARα gene (1387278_at) expression in ‘virtual’ human is twice that of real rat (Fig. 1). Also, the expression of PPARα is greater than the expression of most genes involved in the PPARα pathway. However, reports have shown that PPARα expression in human is 1–10% of the levels found in rodents [28]–[30]. The high level of PPARα gene expression in ‘virtual’ human relative to rat is due to the high specificity of the cross-species ‘virtual’ human strategy. This is made possible by the elimination of poor signals through the selection of highly specific orthologous probes (Fig. 2) for cross-species analysis.

The Affymetrix GeneChip microarray platform uses multiple probes (eleven for the rat microarray) per transcript to estimate the level of expression. If all eleven probes are used to compute the expression level of a cross-species orthologous transcript the non orthologous probes will give a false estimate of expression. By eliminating non orthologous probes via sequence comparison robust estimation of expression of the orthologous transcript is made possible.

### Gene Ranking Analysis using Artificial Neural Networks

Two data sets for the compound EMD were used for ANNs analysis. One consisted of 18 samples (CEL files) of real rat gene expression data and the other 18 samples (CEL files) of virtual human gene expression data. All samples for both data sets were treated identical prior to the generation of CEL files. RMA expression signals for real rat were computed with Affymetrix Rat Genome 230 2.0 library file whilst RMA expression signals for ‘virtual’ human were computed with the rat/human cross-species library file.

Prior to ANNs analysis the data was split into two classifiers, 0 for control and 1 for treated sample. The data was further randomly divided into three subsets; 60% for training, 20% for validation (to assess model performance during the training process) and 20% for testing (to independently test the model for data completely blind to the model). Learning proceeds using the training data and is stopped when the error of the network fails to increase beyond a predetermined number of cycles. Once this is complete the model is then validated on the remaining 20% of the data set aside for independent validation. This process is known as random sample cross-validation which enables the generation of confidence intervals for the prediction of samples.

**Figure 3:**
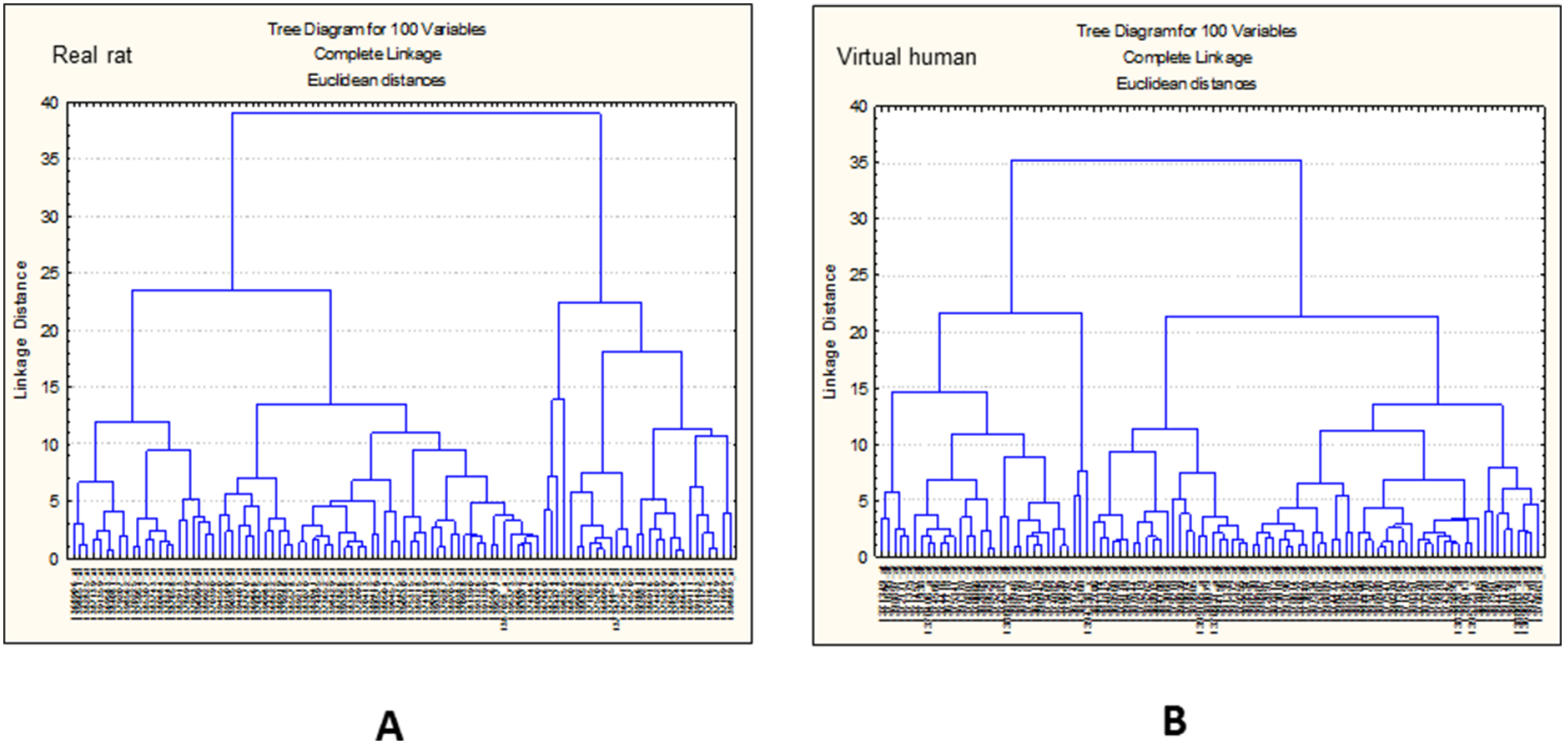
Gene cluster analysis. Hierarchical clustering of real rat (**A**) and virtual human (**B**) 100 top ranked transcripts selected after ANNs analysis.

Detailed examination of the ranked model performance based on the predictive error identified 100 most predictive transcripts for each data set. Hierarchical clustering of each of these 100 sets (real rat and ‘virtual’ human) was performed to identify differences and similarities in the expression profiles (see Fig. 3).

**Figure 4:**
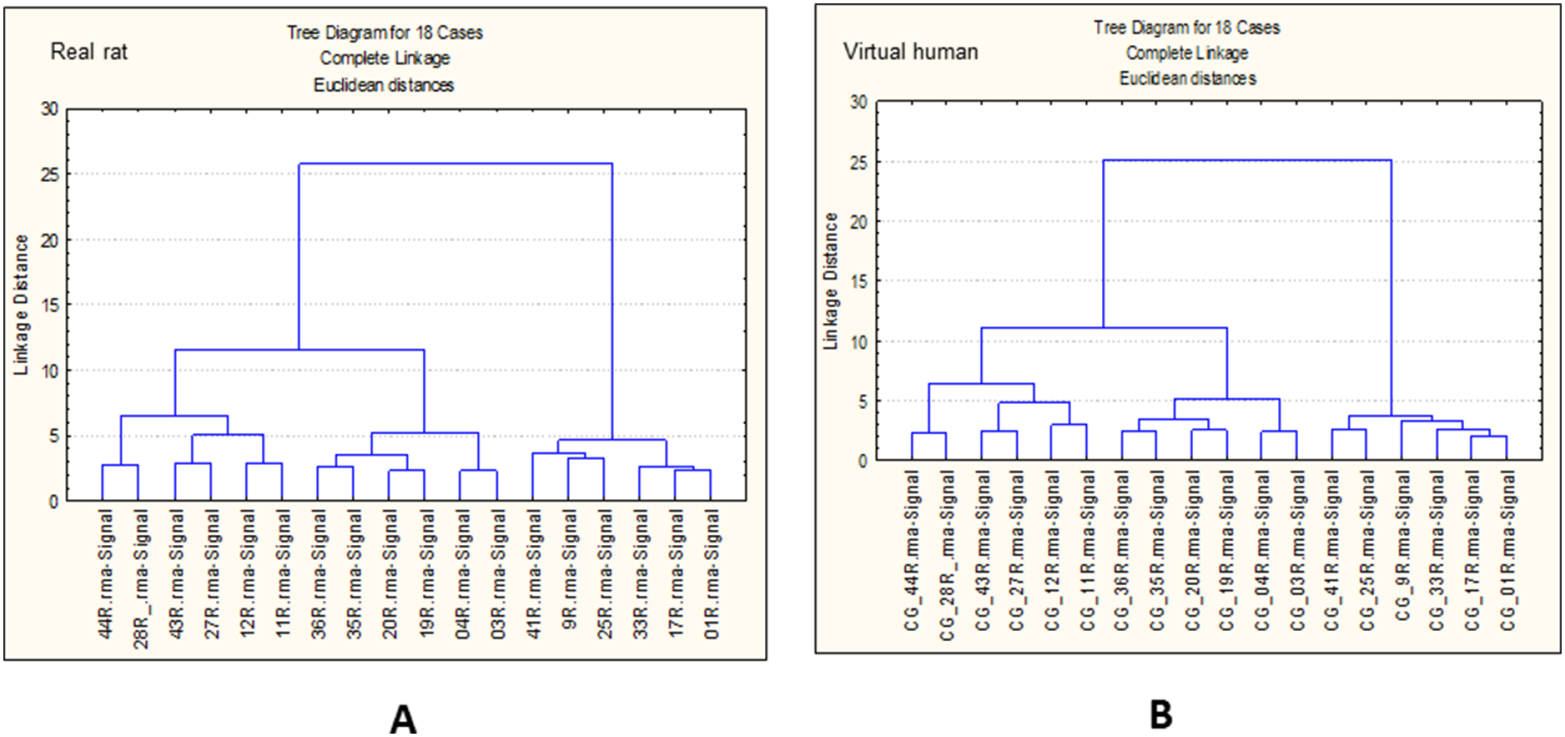
Sample cluster analysis. Hierarchical clustering of real rat (**A**) and virtual human (**B**) samples (CEL files) using the 100 top ranked transcripts selected after ANNs analysis.

In order to assess any effect of the cross-species technology inherent in the ‘virtual’ human data, it was useful to investigate any technical influence that the virtual human library file may be exerting on the data. Therefore hierarchical clustering of samples was performed (see Fig. 4).

Figure 4 suggests that both sets of samples (CEL files for rat and converted CEL files for ‘virtual’ human) were treated identically. In fact the same rat CEL files were made compatible with the cross-species ‘virtual’ human library file in order to generate the ‘virtual’ human RMA condensed hybridisation signals of gene expression. Figure 4 also confirms that the differences in expression profiles observed in Fig. 3 are true biological differences and therefore not a consequence of technical variability imposed by the cross-species technology.

### Analysis of Marker Interactions using Artificial Neural Networks

**Figure 5:**
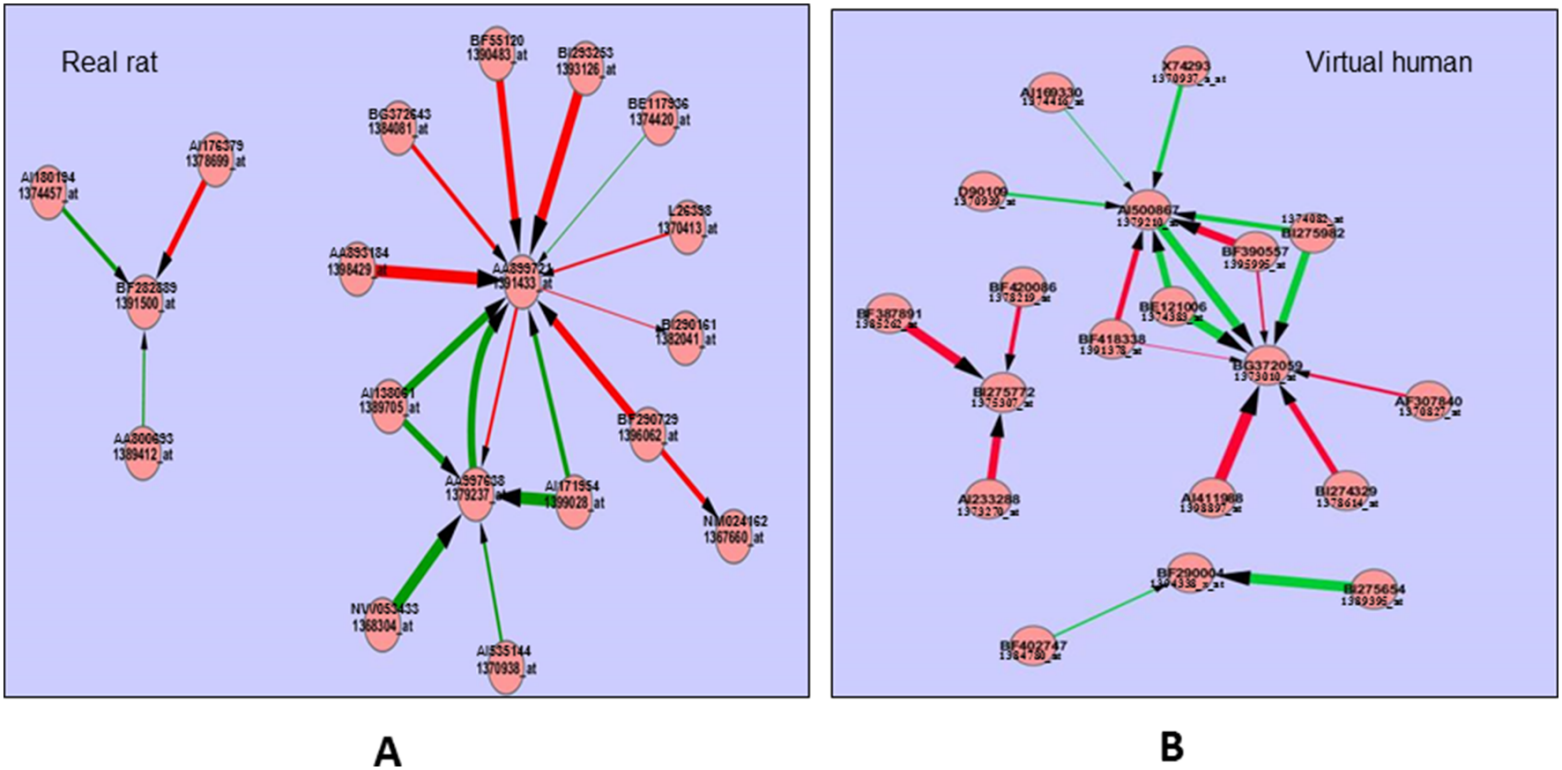
Interactions of putative marker genes. Interactions between 20 putative markers of real rat (**A**) and virtual human (**B**) of the 100 top ranked transcripts after ANNs analysis. Red arrows depict negative interaction whilst green arrows depict positive interactions. The thickness of the arrow indicates the magnitude of the interaction.

ANNs was employed to identify specific markers for EMD which may be responsible for the classification of certain outcomes and in identifying the influence of interacting factors between these markers. Another advantage of ANNs is that they do not rely on any pre-determined relationship between transcripts, meaning, each individual transcript is not initially assumed to be interacting in a biological manner with any other [31]. A pathway distiller tool [22] developed by ANNs was employed to discern the relationship between the 100 top ranked markers for EMD 392949 in both data sets (Table 1). The diagram in Figure 5 depicts the relationships of 10 most negative and 10 most positive interactions between the markers.

**Table 1:** Most predictive virtual human transcripts of EMD activity identified by ANNs analysis.

Probe Set ID	Gene Symbol	Gene Title
1367660_at	Fabp3	fatty acid binding protein 3, muscle and heart
1367718_at	Chkb	choline kinase beta
1367742_at	Cpt1b	carnitine palmitoyltransferase 1b, muscle
1367915_at	Dgat1	diacylglycerol O-acyltransferase 1
1368560_at	Kcnj5	potassium inwardly-rectifying channel, subfamily J, member 5
1368915_at	Kmo	kynurenine 3-monooxygenase (kynurenine 3-hydroxylase)
1369665_a_at	Il18	interleukin 18
1370066_at	Keap1	Kelch-like ECH-associated protein 1
1370406_a_at	Cd55	Cd55 molecule
1370827_at	Cyb5r4	cytochrome b5 reductase 4
1370937_a_at	Itga7	integrin, alpha 7
1370939_at	Acsl1	acyl-CoA synthetase long-chain family member 1
1371349_at	Col6a1	collagen, type VI, alpha 1
1371375_at	Dstn///Dstnl1	destrin///destrin-like 1
1371457_at	---	---
1371492_at	Apobec2	apolipoprotein B mRNA editing enzyme, catalytic
1371695_at	Tpr	translocated promoter region, nuclear basket protein
1371938_at	Caprin1	cell cycle associated protein 1
1372091_at	Mid1ip1	MID1 interacting protein 1
1372171_at	Phc1	polyhomeotic homolog 1 (Drosophila)
1373010_at	Krt12	keratin 12
1373270_at	Wipi1	WD repeat domain, phosphoinositide interacting 1
1373469_at	Ptplad1	protein tyrosine phosphatase-like A domain containing 1
1373648_at	LOC681849	similar to Protein C6orf142 homolog
1374082_at	Ndufaf7	NADH dehydrogenase (ubiquinone) complex I
1374342_at	Ly6g6c	lymphocyte antigen 6 complex, locus G6C
1374383_at	---	---
1374416_at	Coa4	cytochrome c oxidase assembly factor 4
1374471_at	LOC498972	similar to copine II
1374487_at	Fam96a	family with sequence similarity 96, member A
1374566_at	---	---
1375239_at	---	---
1375307_at	Cbx6	chromobox homolog 6
1375341_at	Tmem189	transmembrane protein 189
1375697_at	Mlec	malectin
1376576_at	Dusp11	dual specificity phosphatase 11 phosphatase-like
1376663_at	Dcaf15	DDB1 and CUL4 associated factor 15
1376801_at	RGD1564450	RGD1564450
1377541_at	---	---
1378219_at	Sgtb	small glutamine-rich tetratricopeptide repeat (TPR)
1378614_at	---	---
1378674_at	En2	engrailed homeobox 2
1379178_at	---	---
1379210_at	---	---
1379255_at	Atp6ap2	ATPase, H+ transporting, lysosomal accessory protein 2
1379970_at	Epha4	Eph receptor A4
1380079_at	Samd9l	sterile alpha motif domain containing 9-like
1380351_at	---	---
1382786_at	---	---
1382885_at	Ebf4	early B-cell factor 4
1383068_at	Dtymk	deoxythymidylate kinase (thymidylate kinase)
1383928_a_at	LOC688310	similar to CG5500-PA
1384042_at	---	---
1384360_at	Casz1	castor zinc finger 1
1384780_at	Cpne4	copine IV
1385262_at	---	---
1385534_at	Ngfrap1	nerve growth factor receptor (TNFRSF16) associated protein 1
1386852_x_at	LOC100360645	ubiquitin B-like ubiquitin B-like ubiquitin-40S ribosomal prot
1386988_at	Deaf1	DEAF1 transcription factor
1387015_at	LOC100909840	profilin-2-like///profilin 2
1387358_at	Arl1	ADP-ribosylation factor-like 1
1387561_at	Vipr1	vasoactive intestinal peptide receptor 1
1387644_at	Btc	betacellulin
1387812_at	Pcsk6	proprotein convertase subtilisin/kexin type 6
1387934_at	Bcan	brevican///brevican core protein-like
1388564_at	Kansl2	KAT8 regulatory NSL complex subunit 2
1388603_a_at	Isca1	iron-sulfur cluster assembly 1 homolog (S. cerevisiae)
1388629_at	Impdh2	IMP (inosine 5′-monophosphate) dehydrogenase 2
1389199_at	RGD1309079	similar to Ab2-095
1389395_at	Sepn1	selenoprotein N, 1
1389905_at	---	---
1390230_at	Mip	major intrinsic protein of lens fiber
1390270_at	---	---
1390339_at	---	---
1390413_at	RGD1310371	similar to RIKEN cDNA 1700026D08
1390437_at	Sema5a	sema domain, seven thrombospondin repeats
1390902_at	---	---
1390939_at	---	---
1390942_at	Peli2	pellino E3 ubiquitin protein ligase family member 2
1391314_at	Kcnq5	potassium voltage-gated channel, KQT-like subfamily
1391339_at	---	---
1391363_at	---	---
1391378_at	---	---
1391448_at	Crim1	cysteine rich transmembrane BMP regulator 1 (chordin like)
1393196_at	Klhl23	kelch-like family member 23
1393280_at	Ly86	lymphocyte antigen 86
1393492_at	---	---
1394156_at	Igsf3	immunoglobulin superfamily, member 3
1394270_at	Mks1	Meckel syndrome, type 1
1394338_x_at	Ptk2	protein tyrosine kinase 2
1394416_at	Prickle2	prickle homolog 2 (Drosophila)
1394496_at	---	---
1395323_x_at	---	---
1395995_at	---	---
1397372_at	LOC100910520	MOB kinase activator 1A-like///similar to Mob4B protein
1398449_at	---	---
1398889_at	Polr2m	polymerase (RNA) II (DNA directed) polypeptide M
1398897_at	LOC100912618	ubiquitin-conjugating enzyme E2 variant 1-like
1398946_at	Mrps16	mitochondrial ribosomal protein S16
1399165_a_at	Ccdc97	coiled-coil domain containing 97

Figure 5 demonstrates that different sets of markers can be identified for EMD in real rat and ‘virtual’ human. Importantly this demonstrates the feasibility of identifying bridge effect markers for cross-species extrapolation using cross-species technology. In fact one of the markers, acyl-CoA synthetase (D90109, Fig. 3B), identified with ‘virtual’ human data, is a marker enzyme for PPARα activity [32]. D90109 is also ranked (19) very high in the list of 100 top predictors for ‘virtual’ human but was not selected by ANNs analysis for real rat.

### Analysis of Real and Virtual Human Data

Table 2 depicts fold change data for PPARα pathway genes of real and virtual human in comparison with real rat. There are significant differences in fold changes between virtual human and real human. The marker genes, acyl CoA oxidase (Acox), carnitine palmitoyltransferase I (Cpt1), and hydroxyacyl-CoA dehydrogenase (Hadha) are all down regulated in real human compared to virtual human. These genes are involved in hepatic fatty acid oxidation. The data suggests that elevated levels of expression was due to high levels of PPARα trascripts induced by the EMD392949 agonist.

**Table 2:** Fold change data for PPAR alpha pathway genes for real rat, virtual human and real human.

Gene Symbol	Gene Name	Real Rat^1^	Virtual Human^1^^,^ *	Real Human^1^
Fabp3	fatty acid binding protein 3, muscle and heart	44.66	45.42 (9)	−1.89
Cd36	CD36 molecule (thrombospondin receptor)	5.95	5.92 (10)	4.59
Cpt1b	carnitine palmitoyltransferase 1b, muscle	33.63	33.93 (9)	1.15
Cpt1a	carnitine palmitoyltransferase 1a, liver	5.12	5.13 (10)	1.60
Acsl3	acyl-CoA synthetase long-chain family member 3	7.00	10.35 (6)	1.12
Ehhadh	enoyl-CoA, hydratase/3-hydroxyacyl CoA dehydrogenase	34.08	33.62 (11)	−1.09
Ucp2	uncoupling protein 2 (mitochondrial, proton carrier)	8.53	8.66 (8)	−1.18
Fabp1	fatty acid binding protein 1, liver	10.77	10.77 (10)	2.00
Acox3	acyl-CoA oxidase 3, pristanoyl	1.35	1.34 (8)	−1.21
Hadha	hydroxyacyl-CoA dehydrogenase	3.17	3.15 (10)	1.48
Fabp5	fatty acid binding protein 5, epidermal	1.27	1.28 (9)	−1.06
Acsl1	acyl-CoA synthetase long-chain family member 1	7.57	7.58 (11)	1.30
Cpt2	carnitine palmitoyltransferase 2	3.60	3.5 (9)	1.48
Acsl1	acyl-CoA synthetase long-chain family member 1	14.55	14.31 (9)	1.55
Slc25a20	solute carrier family 25 (carnitine/acylcarnitine translocase)	4.14	4.09 (8)	0.66

*Data in parenthesis indicate number of orthologous probes selected for the virtual human ortholog fold change.

1 Fold change values were calculated at 100 µM of compound.

**Figure 6:**
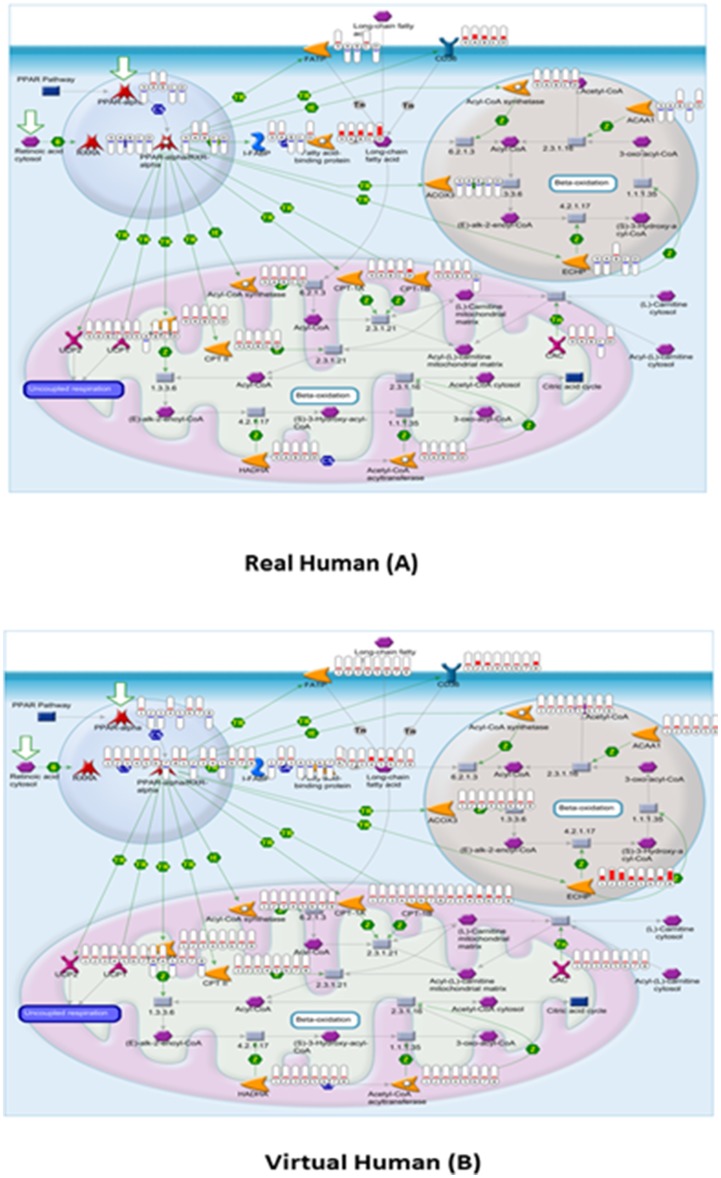
GeneGo MetaCore pathway analysis. MetaCore PPARα pathway mapping of fold change data for real human (A) and virtual human (B). Significant reduction in gene activity of peroxisomal enzymes can be observed for real human (A). Significant fold difference of peroxisomal genes between real and virtual human can be observed, particularly for ACOX3, see text for details. Thermometer-like icons represent samples where red depicts upregulation and blue depicts down regulation.

PPARα is predominatly expressed in tissue capable of fatty acid oxidation such as liver, heart, muscle and brown adipose tissue (Dongiovanni and Valenti, 2013). The most well studied peroxisomal marker enzyme, ACOX, had a mean fold change of −1.21 for real human and 1.34 for virtual human at 100 µM of compound (Fig. 6). The other peroxisomal enzyme, EHHADH, was significantly upregulated (33.62 at 100 µM) in virtual human compared to a mean of −1.09 fold in real human. The most dramatic fold difference was FABP3 with 45.42 fold at 100 µM for virtual human compared to −1.89 fold for real human at 100 µM. PPARα expression stimulates the cellular uptake of fatty acids by increasing the expression of fatty acid binding proteins (FABP) and translocase (Slc25a20).

Given the high homology (see Table 2) of PPARα regulated genes between human and rat the significant fold change differences between virtual human and real human is surprising. For example, all eleven probes for Ehhadh and Acsl1 (see Table 2) of the Affymetrix Rat GeneChip microarray were selected as orthologs for the human orthologous transcripts (virtual human). However, there were dramatic fold change differences between virtual human and real human for the same transcripts. Each Rat GeneChip probe is 25 base pairs, selected within 600 base pairs at the 3′ end of the target sequence [23]. It is therefore conceivable that homology to human is restricted to less than 600 base pairs of the human ortholog. However, this does not fully explain the dramatic fold change differences between virtual human and real human. The weak induction of PPARα regulated genes could be due to the limiting effect of human hepatocytes in culture [9].

### Redundant Probe Sets

In order to deal with splice variance, Affymetrix designed probe sets that can detect several variances of transcripts from a gene leading to a high level of probe set redundancy. For example, there are nine probe sets (1558631_at, 1560981_a_at, 206870_at, 210771_at, 223437_at, 223438_s_at, 226978_at, 237142_at, 244689_at) detecting transcripts of PPARα on the Affymetrix human (U133 Plus 2.0) GeneChip array.

There is no standard way to deal with data from redundant probe sets. Some analysis use the average signal of all probe sets representing the same gene while others (MetaCore) focus on the probe set showing the most differential expression, regardless of the behaviour of other probe sets representing the same gene. Redundant probe sets therefore create bias in function category-based analysis such as pathway and gene enrichment analysis (see below). For most analyses, a one probe set-to-one target relationship will be highly desirable.

This cross-species microarray methodology handles probe set redundancy more effectively in a one-to-one relationship. For example, the virtual human data uses one probe set (1387278_at) to interrogate the PPARα transcript. To achieve this we select orthologous probes by comparing GeneChip probes to cDNA database sequences. This excludes probes that match to non-transcribed regions of the genome.

Ideally, a gene specific probe set should only contain probes whose sequence will be present on the common sequences of all spliced products from the same gene. This means pooling all probes targeting the same gene into one gene specific probe set. For some genes more than eleven probes per probe set will be required to create a gene specific probe set. We believe the gene-based probe set definition is essential for evaluating the overall transcription activity of a gene. Potential alternative splicing events can conceivably be explored by these gene-base probe sets.

### Metacore Pathway Enrichment Analysis

An enrichment analysis will determine which pathways and biological functions are represented in both real and virtual human data. The enrichment analysis takes advantage of novel GeneGo ontoglogies and other publicly available ontologies in MetaCore. Table 3 represents ten enriched pathways in real human and virtual human ranked according to their p-values. Pathway enrichment analysis provides the opportunity to functionally interpret gene expression changes between virtual human and real human.

**Table 3:** Pathways significantly enriched in real human and virtual human hepatocytes.

Pathways significantly enriched in real human hepatocytes	
GeneGo Pathway Map	Real human, −log (pvalue)
Cytoskeleton remodeling TGF, WNT and cytoskeletal remodeling	3.57×10^−27^
Cytoskeleton remodeling	4.98×10^−22^
Cell adhesion, chemokins and adhesion	1.88×10^−20^
Development regulation of epithelia-to-mensenchymal transition (EMT)	7.93×10^−19^
Transport clathrin-coated vesicle cycle	5.22×10^−18^
Development WNT signaling pathway	3.20×10^−17^
Stellate cells activation and liver fibrosis	9.52×10^−17^
Development TGF beta receptor signaling	1.49×10^−14^
Immune response IL-1 signaling pathway	2.07×10^−14^
Signal transduction JNK pathway	4.25×10^−14^
**Pathways significantly enriched in virtual human hepatocytes**	
**GeneGo Pathway Map**	**Virtual human, −log (pvalue)**
Cytoskeleton remodeling TGF, WNT and cytoskeletal remodeling	4.64×10^−27^
Cytoskeleton remodeling	6.38×10^−24^
Cell adhesion, chemokins and adhesion	7.48×10^−17^
Stellate cells activation and liver fibrosis	2.24×10^−15^
Development regulation of epithelia-to-mensenchymal transition (EMT)	5.72×10^−15^
Cell adhesion tight junctions	6.10×10^−15^
NRF2 regulation of oxidative stress response	1.11×10^−14^
Development of EGFR signaling pathway	1.25×10^−14^
Development of TGF beta dependent induction of EMT via MAPK	1.50×10^−14^
Transport clathrin-coated vesicle cycle	1.90×10^−14^

The pathways shown above were all significantly enriched at FDR <0.05 in both species to obtained unadjusted −log (pvalue).

Among the pathways that were enriched in both real human and virtual human, pathways indicative of hepatotocity [7] were strongly represented in both. These include, cytoskeletal remodelling, EMT, stellate cell activation and liver fibrosis. There is some divergence in the type of pathways enriched between real human and virtual human. The pathways specific to virtual human were representative of tumor formation and toxicity. For example, epidermal growth factor (EGFR) signalling, enriched in virtual human, has been implicated in the development of many human cancers and cardiotoxicity [33].

The nuclear factor E2-related factor 2 (Nrf2) is a transcription factor that responds to oxidative stress. The NRF2 pathway has been identified as having regulatory functions in mitochondrial biogenesis, adipocyte differentiation and liver metabolism [34]. The NRF2 pathway was significantly enriched in virtual human but not in real human. Activation of Nrf2 increases energy metabolism and conversely suppresses lipid synthesis. Also, Nrf2 activation may act together with PPARα activation as hepatoprotective response to toxic injury in the liver [35].

Although many of the enriched pathways were common to real human and virtual human there is divergence of significant pathways. Those divergent or less enriched in real human may be due to the variability caused by the hepatocyte culture. It is conceivable that the orthologous probe selection strategy employed for virtual human has reduced some of the genetic variability.

## Conclusions

A single cause for the weak response of PPARα agonists in non rodents is unlikely. For example, although humans have a lower constitutive expression of PPARα [36] than in rodents, it was observed (Fig. 1 & Fig. 2) in this manuscript that, the expression of PPARα in ‘virtual’ human is twice that of real rat. The homology between the DNA binding domain and the ligand binding domain of rat and human PPARα is high [37]. This manuscript found that there is only a 25 base pair difference between rat and human PPARα at the 3′ end. However, human hepatocytes display weak induction of PPARα marker enzyme activity [9], [10], [38].

Significant fold change differences were observed for PPARα regulated genes between real human and virtual human (see Table 2). It appears that there is high sequence conservation of PPARα regulated genes between rat and human, however, gene expression changes do not correspond to the observed sequence conservation. This may be due to the limited expression of PPARα in primary human hepatocyte culture. Primary hepatocyte culture condition can sometimes lead to loss of function of liver specific genes [16]–[18]. Also, primary human hepatocyte cultures are limited by inter-individual variability and short term in vitro life span which does not allow for long term study of PPAR agonists in primary human hepatocyte cultures [9].

Virtual human (orthologs from rat) do not suffer any cell culture effects, contrary to human hepatocytes in culture. This is because gene expression levels (expression estimates) for virtual human were derived from human orthologs of rat hepatocyte cell culture. The cross-species methodology of virtual human therefore attempts to mimic a wild type hepatocyte environment such that gene expression estimates approach wild type estimates.

Further work will be required in order to ascertain how close virtual human expression estimates derived from rat hepatocyte culture approach wild type levels in human. This is beyond the scope of the current work since human hepatocytes (real human) in culture is limited in the PPARα response. The aim of the current manuscript is to demonstrate the utility of the cross-species methodology of virtual human, in that, PPARα activity is significantly different between virtual human and real human hepatocytes.

An efficient and reproducible in vitro system is required for the investigation of the species specificity of the PPARα response. Human hepatocytes have been shown [9], [10], [12], [20], [38] and also in this manuscript, to limit the PPARα response. The work by Mogilengo et al. [13] provides the strongest evidence yet of conserved regulation of PPAR alpha target genes across species via a PPRE-dependant mechanism. Using human hepatoma cells and mouse liver, Mogilengo et al. [13] demonstrated that the regulation of the PPAR alpha target gene, C3 of the complement system, is conserved between human and mouse. This further suggests that, human hepatocyte culture appears to limit the PPAR alpha response. Our manuscript provides further evidence, using a novel cross-species methodology, that human hepatocyte in culture may not be suitable for the investigation of species differences in the PPAR alpha response.

Our cross-species gene expression methodology provides a way of identifying human orthologs of the PPARα response and others, and utilising them in an in vitro system, to elucidate the species specificity that confounds the extrapolation of cross-species preclinical data. Also, the capability to elucidate the molecular mechanism underlying species-specificity of drug-metabolizing enzymes and the utility of identifying bridge effect markers with a cross-species strategy, to assist cross-species extrapolation, was demonstrated.

Furthermore, this cross-species strategy, where human orthologous probes of model species are used for gene expression investigation, in an attempt to mimick the in vivo environment, as demonstrated, provides a suitable model to investigate the efficacy and toxicity of drug candidates on organ systems.

## Supporting Information

Data S1 **Raw data of all rat and virtual human probe set information after ANNs analysis.** This also includes data on error values used to determine ranking order of transcripts. Column A depicts ranking order of rat transcripts and column T depicts ranking order for virtual human transcripts.
(ZIP)
